# Molecular-Targeted Therapies for Epidermal Growth Factor Receptor and Its Resistance Mechanisms

**DOI:** 10.3390/ijms18112420

**Published:** 2017-11-15

**Authors:** Toshimitsu Yamaoka, Motoi Ohba, Tohru Ohmori

**Affiliations:** Institute of Molecular Oncology, Showa University, 1-5-8 Hatanodai, Shinagawa-ku, Tokyo 142-8555, Japan; moba@pharm.showa-u.ac.jp (M.O.); ohmorit@med.showa-u.ac.jp (T.O.)

**Keywords:** epidermal growth factor receptor, resistance mechanisms, cancer

## Abstract

Cancer therapies targeting epidermal growth factor receptor (EGFR), such as small-molecule kinase inhibitors and monoclonal antibodies, have been developed as standard therapies for several cancers, such as non-small cell lung cancer, colorectal cancer, pancreatic cancer, breast cancer, and squamous cell carcinoma of the head and neck. Although these therapies can significantly prolong progression-free survival, curative effects are not often achieved because of intrinsic and/or acquired resistance. The resistance mechanisms to EGFR-targeted therapies can be categorized as resistant gene mutations, activation of alternative pathways, phenotypic transformation, and resistance to apoptotic cell death. Analysis of the processes that modulate EGFR signal transduction by EGFR-targeted inhibitors, such as tyrosine kinase inhibitors and monoclonal antibodies, has revealed new therapeutic opportunities and has elucidated novel mechanisms contributing to the discovery of more effective anticancer treatments. In this review, we discuss the roles of EGFR in cancer development, therapeutic strategies for targeting EGFR, and resistance mechanisms to EGFR-targeted therapies, with a focus on cancer therapies for individual patients.

## 1. Introduction

The epidermal growth factor (EGF) receptor (EGFR) family comprises several isoforms, including ERBB2/HER2, ERBB3/HER3, and ERBB4/HER4 [1]. When the EGFR extracellular domain binds to its ligands, such as EGF and transforming growth factor-α (TGF-α), it forms dimers with other EGFR family members and leads to autophosphorylation of tyrosine residues, thereby activating several downstream signaling pathways, e.g., protein kinase B (AKT/PKB) and mitogen-activated protein kinase (MAPK) pathways, which regulate cell proliferation, survival, and apoptosis [2,3,4]. The constitutive activation of EGFR by gene mutations, gene amplification, or both, has been shown to be related to cancer initiation, progression, and poor prognosis in several cancers, including non-small cell lung cancer (NSCLC), colorectal cancer (CRC), squamous cell carcinoma of the head and neck (SCCHN), and glioblastoma [4,5]. EGFR-targeted inhibitors, including tyrosine kinase inhibitors (TKIs) and monoclonal antibodies (mAbs), are currently being developed and have been approved for use in the treatment of NSCLC, CRC, and SCCHN. These various EGFR-targeted therapies have shown success in different contexts. However, it is difficult to cure patients of NSCLC, CRC, or SCCHN using EGFR TKIs or anti-EGFR antibodies, even when combined with chemotherapy, because tumors inevitably develop acquired resistance to EGFR-targeted therapies [6,7,8].

In this review, we discuss the roles of EGFR in cancer development, therapeutic strategies for targeting EGFR, and the resistance mechanisms to EGFR-targeted therapies (EGFR TKIs and anti-EGFR mAbs), with a focus on cancer therapies for individual patients.

## 2. The Roles of EGFR/ERBB in Cancer

Within the last several decades, numerous studies have shown that EGFR family members play pivotal roles in the occurrence and development of cancer. Genetic alterations in *EGFR* family genes, including point mutations, deletions, and gene amplifications, have been identified in a variety of tumors. Additionally, the improved understanding of the signaling mechanisms and molecular structure of EGFR/ERBB has led to the discovery of molecular-targeted therapies for patients harboring *EGFR* gene alterations. Conversely, clinical implementation of EGFR inhibitors has provided important insights into the mechanisms of cancer development induced by *EGFR*-activating mutations [9]. A series of studies on EGFR, detailed below, has proven the importance of EGFR in preclinical and clinical sciences, thus establishing a basis for cancer therapeutics.

### 2.1. History of Discovery

In 1984, two papers showed that EGFR family members were linked to cancer pathogenesis. Downward et al. found high homology between human EGFR and v-ERBB, a protein from avian erythroblastosis virus that transforms chicken cells [10]. Additionally, Schechter et al. isolated the Neu oncogene from rat neuroblastomas induced by ethylnitrosourea and found that Neu was a rat *Erbb2* gene with a single point mutation in the transmembrane domain [11]. These two studies provided valuable insights into the molecular mechanisms of cancer occurrence caused by aberrations in receptor tyrosine kinases. Subsequently, several overexpression experiments utilizing cultured cells and transgenic mice indicated that EGFR family proteins promote cell proliferation, transformation, and metastasis [12]. For example, EGFR or human EGFR2 (HER2) overexpression induces cellular transformation in NIH3T3 cells [13]. Wild-type HER2 transgenic mice driven by a mammary-specific promoter develop large mammary tumors with metastatic properties [14]. Subsequently, many mutation analyses of patient specimens have demonstrated *EGFR* gene amplification in various human tumors, including lung, head and neck, esophageal, and colorectal cancers [15]. *HER2* amplification and overexpression were also detected in breast, gastric, esophageal, bladder, cervix, salivary duct, and pancreas cancers, as well as glioblastoma [16,17]. These studies provided strong evidence that EGFR/HER family members act as oncogenes in various types of cancer cells.

In 2004, two different groups identified the presence of somatic mutations in the tyrosine kinase domain of EGFR in patients with NSCLC responding to the EGFR TKI gefitinib [9,18]. These somatic mutations were associated with in vitro sensitivity to gefitinib. Interestingly, the response to gefitinib was associated with several clinicopathological features, including Asian ethnicity, female sex, adenocarcinoma histology, and never smoking status [19]. These somatic mutations mainly target exons 18–21 of EGFR, encoding the TK domain, and are clustered around the ATP-binding pocket. The most common and well-characterized EGFR mutations are in-frame deletions in exon 19 (residues 747–750) and the L858R substitution in exon 21, which together account for approximately 80–90% of all the EGFR mutations in NSCLC. These mutant kinases exhibit reduced affinity for ATP, accounting for the increased sensitivity to EGFR TKIs when compared with the wild-type counterparts, as these inhibitors compete with ATP for binding to the catalytic site [20].

### 2.2. EGFR/ERBB Signaling

EGFR/ERBB activation is triggered by the binding of ligands to the extracellular domains of monomeric EGFR, HER3, and HER4. Subsequently, the ligand-binding receptor undergoes dynamic conformational changes, homo-/heterodimer formation, and tyrosine kinase activation [21]. EGFR/HER dimers autophosphorylate the tyrosine residues in the C-terminal tail and the kinase domain, leading to the recruitment and docking of various signaling modules containing the Src homology 2 domain, including kinases, adaptor proteins, ubiquitin ligases, and transcriptional factors [15]. Binding of these signaling molecules to EGFR activates downstream signaling pathways, including the RAS/RAF/MAPK kinase (MEK)/extracellular signal-regulated kinase (ERK), phosphatidylinositol 3-kinase (PI3K)/AKT/mammalian target of rapamycin, SRC, phospholipase C γ/protein kinase C, and Janus kinase (JAK)/signal transducer and activator of transcription (STAT) pathways. Eventually, these signaling pathways induce diverse responses, including activation of cell proliferation and cell motility, promotion of angiogenesis and survival, and inhibition of apoptosis.

HER2 cannot bind any of the known EGFR ligands due to the lack of a ligand-binding domain. However, the extracellular domain of HER2 exhibits a constitutively active conformation, preferably forming heterodimers with other EGFR family members [22]. Once HER2 dimerizes, it exerts the strongest kinase activity among all four EGFR family proteins. Notably, HER2/HER3 dimers have remarkable stability and induce robust downstream signaling in a variety of cancer cells [21]. In addition, HER2 can function in the form of homodimers when overexpressed.

In contrast, HER3 has weak intrinsic kinase activity [23]. Thus, to fulfill its functions, HER3 must interact with other EGFR members and be transphosphorylated by its interacting partner. HER3 contains nine tyrosine phosphorylation sites in the intracellular domain; these sites serve as docking sites for the p85 regulatory subunit of PI3K (Y1054, Y1197, Y1222, Y1260, Y1276, and Y1289), GRB2 (Y1199 and Y1262), and SHC (Y1328). These six PI3K-interacting sites characteristic of HER3 provoke strong survival signals mediated by the PI3K/AKT pathway. In addition, GRB2 and SHC enhance growth signaling through the MAPK pathway.

Only EGFR and HER4 have tyrosine phosphorylation sites for the non-receptor tyrosine kinase SRC (Y974 in EGFR and Y1128 in HER4) and for ABL (Y992 and Y1173 in EGFR; Y875, Y1056, Y1081, Y1150, Y1188, and Y1242 in HER4) [21]. Activation of these kinases leads to cell growth, cell survival, cell spreading, and the stress response.

### 2.3. EGFR/HER Ligands

Ligand overexpression can also cause hyperactivation of EGFR/HER. EGFR/HER ligands can be classified into three groups: (1) ligands that bind to EGFR specifically (EGF, TGF-α, amphiregulin, and epigen); (2) ligands that bind to both EGFR and HER4 (betacellulin, HB-EGF, and epiregulin); and (3) ligands that bind to HER3 and/or HER4 (the neuregulin (NRG) family, including NRG1–4) [24]. These ligands act as autocrine or paracrine growth factors that are derived from cancer cells or the tumor stroma, respectively. Overproduction of these EGFR ligands is observed in several human tumors. For example, TGF-α is frequently overexpressed together with EGFR in lung, colorectal, breast, ovary, prostate, and head and neck carcinomas, and is associated with a poor prognosis [21,25]. HB-EGF derived from cancer-associated fibroblasts in the stroma promotes the proliferation of uterine cervical cancer cells [26]. NRG1 accelerates the progression of gastric cancer via the self-renewal of cancer stem cells. Rearrangement of the *NRG1* gene is found in breast cancers, independent of HER2 overexpression [27,28]. Wilson et al. reported that autocrine NRG1 drives HER3 activation in a subset of SCCHN. An EGFR/HER2 kinase inhibitor, lapatinib, is highly sensitive to SCCHN expressing NRG1 but is lacking HER2 amplification [29]. Moreover, Yonesaka et al. showed that NRG1 is overexpressed in patients with NSCLC that is resistant to first-generation EGFR TKIs. Afatinib overcomes NRG1-mediated resistance by inhibition of pan-HER family activity [30].

### 2.4. Nuclear Localization and Functions of the EGFR/ERBB Family

EGFR functions not only on the plasma membrane but also in the nucleus. EGFR is downregulated after binding to its ligands, subsequently undergoing degradation or recycling back to the plasma membrane via clathrin-dependent and -independent endocytosis. However, some EGFR undergoing endocytosis is translocated into the nucleus by classical importin α/β-dependent mechanisms [31]. Nuclear EGFR has been observed in various cancers, including breast cancer, NSCLC, and SCCHN [32,33]. Translocated EGFR/ERBB family proteins function as positive regulators of transcription, DNA replication, and DNA repair, resulting in proliferation, angiogenesis, and metastasis of cancer cells [34]. EGFRs activate the transcription of cancer-associated genes, including genes encoding cyclin D1, aurora kinase A, cyclooxygenase-2, inducible nitric oxide synthase, c-Myc, STAT1, and B-Myb, through the C-terminal region as cotranscriptional activators [35,36,37]. In addition, nuclear EGFR phosphorylates proliferating cell nuclear antigen (PCNA) on Y211, leading to increased PCNA stability and cell proliferation. Furthermore, upon exposure to ionizing radiation, oxidative stress, and platinum-based drugs, EGFR is imported into the nucleus and interacts with DNA-dependent protein kinase (DNA-PK), thereby promoting repair of DNA double-stranded breaks [38]. Cetuximab suppresses DNA-PK activity through the inhibition of radiation-induced EGFR nuclear import [39]. Moreover, nuclear EGFR contributes to resistance to radiation, chemotherapy, and EGFR-targeted drugs, such as gefitinib and cetuximab [40].

## 3. Amplifications and Mutations in EGFR in Cancer

### 3.1. EGFR

In glioma, an *EGFR* mutant lacking exons 2–7, corresponding to the EGFR extracellular domain, has been identified [41]. The oncogenic mutation denoted as *EGFRvIII* (EGFR verIII) is found in approximately 40% of high-grade human gliomas (glioblastoma multiforme (GBM)) with wild-type *EGFR* amplification [42]. This mutation has also been found in breast, lung, head and neck, ovarian, and prostate cancers [43,44]. EGFRvIII confers enhanced tumorigenicity through ligand-independent dimerization and constitutive activation, which are both caused by the deletion of the ligand-binding domain [45]. Accordingly, this mutation is considered as a plausible therapeutic target, and the efficacy of specific antibodies, vaccines, and EGFR TKIs has been evaluated in multiple clinical trials, some of which have resulted in regulatory approval (see below) [46]. The specific agents that have been evaluated include radiolabeled mAbs (^125^I-EGFR mAb 425), toxin-conjugated mAbs (ABT414, AMG595), and EGFRvIII-specific peptide vaccines (rindopepimut) [47,48,49,50]. However, the results for these drugs (^125^I-EGFR mAb 425, AMG595, and rindopepimut) have been disappointing. For example, a clinical trial for rindopepimut was terminated in March 2016 owing to no significant prolongation of overall survival [51]. ABT414 is under evaluation, and encouraging results are expected if the associated difficulties can be overcome (ClinicalTrials.gov Identifier: NCT02573324 and NCT02590263).

In addition, EGFRc958, the second most common EGFR mutant, harboring a deletion in amino acids 521–603, occurs together with amplification of wild-type EGFR in ~20% of GBM, resulting in enhanced ligand-dependent kinase activity [52].

Clinical application of EGFR TKIs in NSCLC treatment has led to the discovery of somatic activating mutations in EGFR in a subset of NSCLCs [9,18]. High clinical response to gefitinib or erlotinib has been attributed to five amino acid deletions in exon19 (∆746–750) or amino acid substitution of arginine for leucine at position 858 (L858R) in exon 21. Further studies have revealed an additional point mutation at Gly719 in exon 18, which is substituted with serine, cysteine, or alanine, although less frequently [53,54]. These mutations are clustered around the ATP-binding pocket of the tyrosine kinase domain, resulting in higher activity of EGFR. Additionally, the ∆746–750 and L858R mutants have a higher Km value for ATP than the wild-type EGFR (137- and 6-fold increase, respectively) [55]. A 50-fold increase in activity relative to wild-type EGFR was observed in the L858R mutant due to disruption of the auto-inhibitory machinery of EGFR [20]. The prevalence of *EGFR*-activating mutations is 5–40%, depending on the population; these mutations are detected in up to 10% of Caucasians, whereas 30–40% of East Asian patients have EGFR-activating mutations. These mutations have also been frequently found in patients with adenocarcinoma, women, and never-smokers [56].

EGFR overexpression is observed in 40–80% of patients with NSCLC [57], owing to epigenetic transcriptional activation and gene copy number (GCN) alterations. Notably, selective amplification of mutant alleles is often observed in tumors with the *EGFR* mutations [54].

*EGFR* gene amplification has also been observed in CRC and SCCHN, although *EGFR* mutations are less commonly detected [58]. Several studies using fluorescence in situ hybridization have demonstrated that about 40% of CRC contains *EGFR* amplifications with increased *EGFR* GCN [59]. A subset of CRC also shows *KRAS* mutations with *EGFR* gene amplification. Astrocytomas, anaplastic astrocytomas, and GBM exhibit *EGFR* gene amplification with approximately the same incidence (33%), although large amplifications have only been observed in GBM. Patients with EGFR overexpression have shorter survival times than patients without gene amplification in GBM [60,61].

### 3.2. HER2

In a variety of tumors, HER2 aberrations primarily include gene amplification and protein overexpression. Overall, approximately 1–37% of tumors exhibit HER2 overexpression. In particular, approximately 20% of breast cancers harbor *HER2* amplification, and this mutation acts as a driver mutation to promote breast cancer progression. Therefore, anti-HER2 therapies have provided significantly improved outcomes in patients with HER2-positive breast cancer [17]. Additionally, several studies have reported a relatively high frequency of HER2 overexpression in gastric or gastroesophageal cancers, ranging from 6% to 30% [62]. The rate of HER2 overexpression varies according to histological type, e.g., 15–50% of intestinal-type and 2–25% of diffuse/mixed-type cancers, based on the Lauren classification, showing HER2 overexpression [63]. Highly differentiated gastric cancers with HER2 overexpression exhibit significantly poor prognosis. Moreover, *HER2* amplification was found in 2–29% and 5–15% of patients with pancreas and bladder cancer, respectively.

Somatic mutations in HER2 have been reported in NSCLC, CRC, gastric cancer, and a small subset of ovarian cancer, but not in pancreatic cancer [64]. These mutations include missense mutations, duplications, and small in-frame insertions, e.g., L755P, G776S, ins774 (AYVM), and ins776 (YVMA) [65], leading to increased signaling activity. Mutations in HER2 are almost exclusively found in cancers without *HER2* gene amplification, and the HER2 mutation is more frequently observed in never-smokers [66].

### 3.3. HER3 and HER4

HER3 acts as a signal-promoting molecule via its interactions with HER2 and EGFR, as described above. Recently, however, whole-exome sequencing has revealed several somatic mutations in ~11% of CRC cases and 12% of gastric cancer cases. Most mutations in HER3 are clustered in the extracellular domain (V104, A232, P262, G284 D297, G325, and T355), and two mutations were located in the kinase domain (S846I and E928G). HER3 mutants are required for HER2 to promote oncogenic signaling, acting by inducing anchorage-independent growth and tumorigenesis in a ligand-independent manner [67]. Similarly, mutations in HER4 have been identified in melanoma, NSCLC, and medulloblastoma [68,69].

## 4. Cancer Therapy Targeting EGFR

Given the function of EGFR in diverse cellular processes, two therapeutic approaches, i.e., TKIs and mAbs, are currently employed for targeting EGFR in various human cancers. Each of these approaches has a distinct mechanism; TKIs target the intracellular tyrosine kinase domain, and anti-EGFR antibodies bind to extracellular domains. Several studies have confirmed the significant benefits of EGFR-targeted agents in several types of cancers, including NSCL, CRC, pancreatic cancer, breast cancer, and SCCHN (Table 1 and Table 2). Moreover, overall survival, progression-free survival (PFS), and overall response are prolonged in these cancers [70,71,72,73,74].

### 4.1. TKIs Targeting EGFR

TKIs are small molecules that show reversible or irreversible binding to the receptor. These agents bind to ATP-binding pockets on the intracellular catalytic kinase domain of receptor tyrosine kinases (RTKs), preventing autophosphorylation and activation of several downstream pathways [75]. Reversible inhibitors compete with ATP to recognize the kinase-active conformation, whereas irreversible inhibitors bind to the kinase active site covalently by specifically reacting with a cysteine residue. In addition, irreversible inhibitors have the advantage of prolonging the clinical effects of the drug and decreasing the need for frequent dosing, although specificity and tolerability may be compromised [76]. However, the safety profiles of these targeted strategies should also be considered; skin rashes, such as acneiform eruptions, hyperpigmentation, xerosis, trichomegaly, and paronychia, are often observed. Notably, the immediate side effects that are associated with EGFR inhibitors are generally mild to moderate, and most of the side effects are temporary, with resolution observed after a few weeks. However, in some patients, these side effects can lead to dose reduction or even discontinuation of treatment [77,78,79]. Importantly, pulmonary toxicities, particularly drug induced-interstitial lung disease (ILD), have emerged as critical adverse drug reactions. Less than 5% of patients administered EGFR TKIs develop ILD, and the mortality rate is 0.5–1.5%. The risk factors for ILD in patients undergoing EGFR TKI treatment include a history of smoking, concomitant interstitial pneumonia, and poor performance status [80]. Physicians must cautiously weigh the benefits and risks of targeted therapies associated with ILD to provide optimal treatments and favorable outcomes.

Currently, several EGFR TKIs are clinically available. First-generation EGFR TKIs (gefitinib and erlotinib) exhibit excellent clinical efficacy in patients with NSCLC carrying *EGFR*-activating mutations (exon 19 15 bp del, and exon 21 L858R). Covalent inhibitors can have advantages over reversible inhibitors because they achieve complete and sustained target engagement in the presence of high intracellular concentrations of the competitive ligand ATP, which requires the physical turnover of the targeted protein to restore inhibited signaling pathways. Second-generation covalent inhibitors of EGFR (afatinib and dacomitinib) demonstrate an increased cellular potency against EGFR oncogenic variants (e.g., EGFR-L858R/T790M) [81]. These compounds utilize an aniline moiety to bind in the back pocket, and the aniline moiety clashes with the Met790 side chain, thus reducing the activity of the EGFR-T790M protein compared with that of the wild-type protein. However, the second-generation EGFR-TKIs potently inhibit wild-type EGFR, causing epithelium-based toxicities, such as rash and diarrhea, thereby limiting their clinical dose. To counter T790M-dependent resistance, third-generation covalent EGFR TKIs (osimertinib, olmutinib, and rociletinib) have been developed with a high potency toward T790M-containing mutants and selectivity over wild-type EGFR [82]. Both osimertinib and rociletinib exhibit strong clinical efficacy and were designated as breakthrough therapies by the US Food and Drug Administration (FDA) for treating NSCLC with T790M mutations [83]. However, as with first- and second-generation EGFR TKIs, clinical use of third-generation EGFR TKIs also induces resistance and leads to disease progression. To overcome resistance to first-, second-, and third-generation EGFR TKIs, an understanding of the resistance mechanisms is critical.

Gefitinib, erlotinib, afatinib, and osimertinib are currently clinically approved by the FDA for the treatment of NSCLC, and erlotinib is available for use in pancreatic cancer in combination with gemcitabine. Lapatinib, which is clinically available for patients with breast cancer, is a small molecule that inhibits the tyrosine kinase activities of HER2 and EGFR. In preclinical studies, lapatinib was found to not be cross-resistant with trastuzumab (10–12). Clinical activity of lapatinib in combination with capecitabine has been demonstrated in women with HER2-positive breast cancer that progressed while the patients received trastuzumab. Vandetanib, a reversible ATP-competitive multitarget tyrosine kinase inhibitor that targets RET, vascular endothelial growth factor receptor (VEGFR), and EGFR tyrosine kinases, is clinically available for the treatment of symptomatic or progressive medullary thyroid cancer in patients with unresectable, locally advanced, or metastatic disease. Somatic mutations in RET are also found in 30–50% of patients with medullary thyroid cancer. Analyzing data from preclinical studies and patient tissues will be critical to both fully understand the resistant mechanisms and guide to strategies to overcome resistance.

### 4.2. Anti-EGFR mAbs

mAbs against EGFR were specifically designed against the EGFR extracellular region, resulting in competitive inhibition of ligand binding and the prevention of receptor dimerization, autophosphorylation, and downstream signaling [84]. In addition to inhibiting EGFR, these mAbs induce receptor dimerization, ubiquitination, degradation, and prolonged downregulation [85]. As alternative mechanisms, binding of the mAb may lead to induction of antibody-dependent cell-mediated cytotoxicity, resulting in the induction of endocytosis, and complement-mediated cytotoxicity may occur [86].

Currently, there are two clinically available anti-EGFR mAbs: cetuximab and panitumumab. Cetuximab, an IgG1 human-mouse chimeric anti-EGFR mAb, has activity against metastatic CRC and SCCHN with wild-type KRAS. Panitumumab is a fully humanized IgG2 mAb that is approved for the treatment of metastatic CRC with wild-type KRAS.

## 5. Mechanisms of Resistance to EGFR TKIs

**Figure 1:**
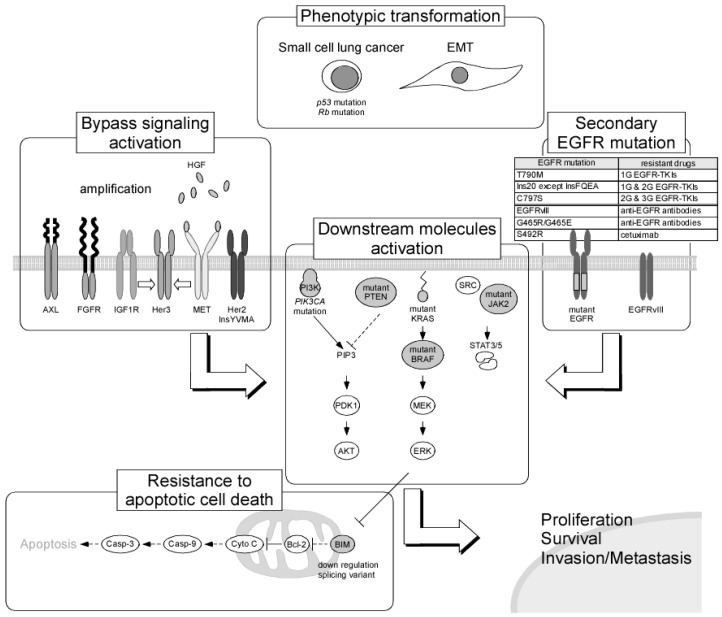
Resistance mechanisms to EGFR tyrosine kinase inhibitors (TKIs) and anti-EGFR antibodies. Abbreviations: AXL, anexelekto; FGFR, fibroblast growth factor receptor; IGF1R, insulin-like growth factor 1 receptor; HGF, hepatocyte growth factor; MET, HGF receptor; PI3K, phosphoinositide 3-kinase; PIK3CA, phosphatidylinositol-4,5-bisphosphate 3-kinase, catalytic subunit alpha; PIP3, phosphatidylinositol (3,4,5)-trisphosphate; PDK1, pyruvate dehydrogenase lipoamide kinase isozyme 1; PTEN, phosphatase and tensin homolog; JAK2, janus kinase 2; STAT, signal transducer and activator of transcription; EMT, epithelial-mesenchymal transition; P53, tumor protein p53; Rb, retinoblastoma tumor suppressor; 1G, 1st generation; 2G, 2nd generation; 3G, 3rd generation; Casp-3, caspase-3; Casp-9, caspase-9; Cyto C, cytochrome c; Bcl-2, B-cell lymphoma 2; BIM, Bcl-2-like 11.

NSCLC harboring EGFR-activating mutations is known to exhibit recurrence after 9–13 months of continuous administration of drugs. The emergence of resistance to anticancer drugs is thought to be the most important problem for achieving effective cancer therapy. For the identification of the optimal therapeutic drugs for patients with cancer, improving the understanding of drug resistance mechanisms is essential, and many preclinical and clinical investigations of drug resistance have been performed. Studies examining resistance to molecular-targeted drugs may provide the most valuable information, although several studies were performed during the early era of cancer chemotherapy, and many resistance mechanisms, including the presence of multidrug resistance genes, have been revealed. In recent years, to achieve the individualization of cancer therapy, whole-genome analyses of cancer tissues using next-generation sequencing have been performed and have provided not only detailed mapping of genomic alterations but also information regarding cancer evolution as it relates to cancer therapy. Exploration of the determinants of sensitivity and resistance to EGFR TKIs can be accelerated by using such novel technologies. Resistance mechanisms to EGFR TKIs can be classified into four groups as follows: secondary mutations in the EGFR gene, activation of alternative pathways, phenotypic transformation, and resistance to apoptotic cell death (Figure 1).

Even in cases of multitargeted kinase inhibitors, such as vandetanib [87], these mechanisms are thought to evade the antitumor effects of the drugs when inhibition of EGFR is the main cause of cytotoxicity. Notably, *EGFR* T790M as a secondary mutation is the most frequent cause of resistance to first-generation EGFR TKIs. To overcome this resistance, third-generation EGFR TKIs were developed with promising effects. For this reason, multiple biopsies are recommended for recurrent NSCLC tumors. In contrast, many molecular-targeted drugs against alternative signaling molecules have been developed, and several combinations of EGFR TKIs with these drugs have been found to exhibit synergistic effects against EGFR TKI-resistant cancer in preclinical studies. However, the clinical efficacy of such combination chemotherapies has not yet been demonstrated.

### 5.1. Secondary Mutations in the EGFR Gene

First-generation EGFR TKIs that bind to EGFR competitively with ATP show high antitumor activity against NSCLC harboring EGFR-activating mutations due to the high affinity to the ATP-binding domain [88]. It is possible that first-generation EGFR TKIs that originally targeted wild-type EGFR could become therapeutic drugs for NSCLC based on this unintended characteristic. However, secondary mutations in *EGFR*, resulting in T790M in exon 20, decrease the binding affinity of first-generation EGFR TKIs to EGFR due to conformational changes and cause resistance [89]. For the T790M mutation, first-generation EGFR TKI binding affinity decreases to the same level as ATP because threonine at the ATP binding pocket is substituted by a larger molecule, methionine [88]. Indeed, NSCLC cells harboring T790M mutant EGFR show more than a 1000-fold resistance to first-generation EGFR TKIs as compared with parental cells, even if they have EGFR-activating mutations [90]. Similar mutations in the ATP-binding pocket have been observed in tumors that are resistant to other RTK inhibitors (e.g., Bcr-Abl T315I, Kit T670IE, EML4-ALK L1196M); these mutations are therefore called gatekeeper mutations. The T790M mutation is found in 50–60% of patients with NSCLC after treatment with first-generation EGFR TKIs [89,91], and this frequency is much higher than that of other gatekeeper mutations. This difference is thought to be related to the binding of first-generation EGFR TKIs to the active form of EGFR, whereas the other inhibitors (imatinib and crizotinib) bind to the inactive form of the target. In a recent report using ultrasensitive digital polymerase chain reaction (PCR), the T790M mutation was found in 79.9% of patients with NSCLC as an *EGFR*-activating mutation before EGFR TKI treatment [92]. Because the frequency of allelic emergence of this mutation is less than 0.1% in most cases, it is thought that first-generation EGFR TKI-responsive tumors acquire resistance by the selection of a minor population cancer cells expressing T790M. To overcome T790M mutation-mediated resistance, second-generation EGFR TKIs (e.g., afatinib, dacomitinib, and neratinib), which irreversibly bind to EGFR, have been developed. Although afatinib was approved as a first-line chemotherapeutic drug for patients with NSCLC harboring the *EGFR*-activating mutation, the clinical dose of this drug cannot reach a concentration that will inhibit T790M mutant EGFR [93,94]. Novel pyrimidine-based third-generation EGFR TKIs have inhibitory effects on activation mutations and the T790M mutation specifically, and do not act on wild-type EGFR. Moreover, the third-generation EGFR TKIs osimertinib and olmutinib have shown high objective responses (ORRs) in 50–60% of patients with the T790M mutation [95,96] and were approved as second-line drugs for resistant tumors after first-generation EGFR TKI administration. Furthermore, in a recent phase Ib/II trial, osimertinib was used as a first-line treatment for advanced NSCLC harboring mutant EGFR, with a robust ORR (67%) and an extremely prolonged PFS of 22.1 months as compared with the first-generation EGFR TKI (8.4–13.1 months) [97]. These results suggest that an initial treatment for predicted resistance mechanisms may be able to prevent the acquisition of resistance and extend the period until emergence of resistance.

Analysis of EGFR mutations related to EGFR TKI resistance using clinical specimens has also identified many relatively rare mutations in exons 18–21, which are near the tyrosine kinase-coding region [98]. For example, exon 20 insertion comprises approximately 6% of *EGFR* mutations. Most of these mutations are related to resistance to first- and second-generation EGFR TKIs, whereas only A763_Y764insFQEA shows sensitivity to first-generation EGFR TKIs [99,100]. C797S in exon 20 was detected in T790M-positive tumors as a “tertiary” substitution mutation at the essential site for the covalent bond with drugs, causing resistance to second-generation and third-generation EGFR TKIs. Interestingly, the singular C797S mutation retains sensitivity to first-generation EGFR TKIs, and the combination of first- and third-generation EGFR TKIs overcomes resistance when C797S and T790M mutations exist in different alleles (trans) [101]. Alternatively, minor mutations, such as E709X, Ins19, Ins20, S681I, and L861Q, have been detected at low rates in clinical specimens and are expected to mediate resistance. Although the contribution of these mutations to EGFR TKI sensitivity has not been completely clarified for rare mutations, some mutations cause drug-specific sensitivity beyond the classification of EGFR TKIs by generation. To determine the optimal drug for patients with NSCLC harboring *EGFR* mutations, it is necessary to obtain data from preclinical experiments, including patient-derived xenograft models. Moreover, the importance of analyzing large datasets based on the accumulation of clinical sensitivity and gene mutation data is expected to increase in the future.

### 5.2. Activation of Alternative Pathways

Conversion from EGFR to activation of alternative signaling pathways is another cause of EGFR TKI resistance. Activation of other RTKs and constitutive activation of downstream molecules are included in these mechanisms and can cause alterations in cellular dependence on proliferative or anti-apoptotic signaling. The most frequently observed resistance in this category is caused by signaling through the hepatocyte growth factor (HGF) receptor MET. MET signaling mediates AKT signaling via HER3 rather than EGFR signaling; therefore, MET activation induces EGFR TKI resistance [102]. Hyperactivation of MET signaling is caused by *MET* gene amplification or by an increased HGF supply via autocrine or paracrine signaling. Emergence of *MET* gene amplification has been reported in approximately 20% of tumors with acquired resistance [103,104], with a prevalence of 4–10% when the cutoff value was set at four copies [105]. In cases of activation by HGF, MET activates downstream signaling pathways via the MET adapter protein, Gab1 [106,107]. In an analysis of a Japanese cohort of NSCLC with the EGFR-activating mutation, a higher expression of HGF was detected in 61% of tumors with acquired resistance to first-generation EGFR TKIs and in 29% of patients who initially showed low sensitivity to the therapy [108]. Even in patients with EGFR TKI-resistant cancer due to MET hyperactivation, EGFR signaling pathways remain active. Therefore, although MET TKI monotherapy does not have antitumor effects, a combination therapy with EGFR and MET TKIs can overcome this resistance [102]. Several clinical trials of combination chemotherapy of MET TKIs (tivantinib, cabozantinib, and INC280) and EGFR TKIs are now being planned and implemented. However, the combination of erlotinib and tivantinib did not show efficacy in patients with NSCLC after EGFR TKI treatment failure [99,109]. Even after combined treatment with EGFR and MET TKIs, extreme acquired resistance occurred in a preclinical model [110]. Therefore, to treat individual patients, studying the development of acquired resistance mechanisms via tissue biopsy or plasma analysis is necessary.

Upregulation of EGFR family members also contributes to EGFR TKI resistance. Amplification of *HER2* is observed in only 1% of chemo-naïve patients with NSCLC, but this proportion increases to 12% in EGFR TKI-resistant tumors, suggesting that the overexpression of HER2 may contribute to resistance [111]. Moreover, several exon 20 insertion mutations in the *HER2* gene, such as EGFR mutations, are known to be found in cancer. Among them, the HER2 in-frame 766–779 YVMA insertion (*HER2* InsYVMA), which is frequently detected in NSCLC [112], induces constitutive activation of this receptor, resulting in downstream signaling activation independently of EGFR [113]. Pan HER family receptor inhibitors, i.e., second-generation EGFR TKIs, show inhibitory effects on NSCLC expressing HER2 with exon 20 insertions [114]; however, effective drugs for HER2 InsYVMA have not yet been developed. For the EGFR/HER2 dual kinase inhibitor lapatinib, both gatekeeper mutations, i.e., HER2 T798I and EGFR T790M, were reported to mediate comparable levels of resistance to this drug [115]. HER3 is known to form heterodimers with other HER family receptors, and HER2/HER3 heterodimers are most frequently involved in cancer development and progression [116]. HER3 is upregulated in a compensatory manner by EGFR TKI exposure due to AKT-mediated negative feedback signaling, which consequently causes EGFR TKI resistance [117].

During the activation of other RTKs, insulin-like growth factor receptor 1 [118], AXL [119], and fibroblast growth factor receptor (FGFR) [120] are known to contribute to bypass signaling. In addition, overexpression of integrin 1, which is related to cell adhesion signaling, mediates EGFR TKI resistance [121]. In particular, many small molecule inhibitors and antibodies have been developed to target these RTKs, and several combination chemotherapies with EGFR TKIs have been evaluated in clinical studies. However, none of these approaches were found to be efficacious in patients with NSCLC who have experienced EGFR TKI treatment failure.

For downstream signaling molecules, the downregulation or deficiency of phosphatase and tensin homolog (PTEN) [122,123] and amplification or E545K mutation of the *PIK3CA* gene, which encodes the catalytic subunit of PI3K [124,125], activate the PI3K/AKT signaling pathway and contribute to EGFR TKI resistance. In the MAPK signaling pathway, the constitutively active form of KRAS was found to contribute to resistance during the initial steps of EGFR TKI development [126]. Moreover, emergence of *BRAF* mutations (V600E and G469A), which is detected in 1% of EGFR TKI-resistant tumor samples, also decreases drug sensitivity [127]. We previously reported that transiently enhanced KRAS expression without gene mutation correlates with afatinib resistance [128].

In a phase I/II study of osimertinib as a first-line treatment for NSCLC, an analysis of circulating tumor DNA in postprogression plasma samples was performed for 38 patients [97]. Circulating tumor DNA was detected in 50% of the samples, and the emergence of putative resistance mechanisms was confirmed, including gene amplifications (*MET*, *EGFR*, and *KRAS*) and other mutations (*EGFR* C797S, *HER2* exon 20 insertion, *MEK1*, *KRAS*, *PIK3CA*, and *JAK2*), although the acquired EGFR T790M mutation was not observed. The development of chemotherapies to target these resistance mechanisms is expected to be a major focus in the future.

### 5.3. Phenotypic Transformation

Transformation to small cell lung cancer (SCLC) is found in some NSCLCs with an acquired resistance to EGFR TKIs [104,129]. These transformed tumors are thought to acquire resistance due to the decreased expression of the mutant EGFR. However, because mutant EGFR expression is still detectable in the transformed tumors [105], the emergence of SCLC is thought to be caused by transformation of NSCLC rather than by de novo clonal appearance. Inactivation of Rb1 and TP53, which is nearly ubiquitously observed in SCLC, also occurs in the transformed tumor. Recently, whole-genome sequencing analysis and immunohistochemical analysis for elucidation of the evolution of resistance were performed in specimens of advanced lung adenocarcinoma (LADC) with EGFR mutations, which subsequently transformed into SCLC after EGFR TKI treatment [130]. As a result, the complete inactivation of both Rb1 and TP53 was observed in LADC before EGFR TKI treatment. Moreover, EGFR mutant LADC harboring complete inactivation of both genes had a 43-fold greater risk of SCLC transformation than other tumors. These observations indicate that EGFR TKI-resistant SCLCs branch out early from LADC clones and are selected by EGFR TKI treatment.

The epithelial-mesenchymal transition (EMT) of tumor cells results in stem cell-like characteristics [131] and causes resistance to many anticancer drugs [132], including EGFR TKIs [133]. Induction of the EMT is known to be related to many factors, including various signaling pathways (TGF-α, Wnt, HGF, GAS6, VEGF, and FGF), hypoxia-inducible factor-1 activation under hypoxic conditions, and inflammatory cytokines, such as prostaglandin E2. All of these factors may induce resistance to EGFR TKIs by activation of the EMT. For example, activation of AXL signaling enhances vimentin expression and causes the EMT [134]. Overexpression of AXL and its ligand GAS6 has been observed in EGFR TKI-resistant tumors, and the activation of this signaling pathway contributes to EGFR TKI resistance through induction of the EMT and activation of bypass signaling [119]. To date, no therapeutic strategies for EMT-mediated EGFR TKI resistance have been developed.

### 5.4. Resistance to Apoptotic Cell Death

EGFR TKIs induce apoptotic cell death rather than cytostatic effects in cancer cells by controlling the expression of Bcl-2 family proteins. The BH3-only pro-apoptotic member of the Bcl-2 family, Bcl-2-like 11 (BIM), is phosphorylated at serine 69 by ERK and is subsequently degraded by the ubiquitin/proteasome pathway. BIM is expressed at a concentration that does not induce apoptosis [135,136]; however, when ERK is inhibited by EGFR TKIs, apoptosis is induced owing to the increased intracellular concentration of BIM resulting from decreased degradation [137,138]. BIM is thought to be a key mediator of apoptosis induction by EGFR TKIs, and tumors with a decreased expression of BIM show resistance to EGFR TKIs [139,140]. Moreover, BIM inhibits other anti-apoptotic Bcl-2 family proteins via its BH3 domain; accordingly, a splicing variant of BIM lacking the BH3 domain also causes resistance, and this splicing variant is specifically observed in Asians (10.9–18.6%). Moreover, PFS is significantly shortened in EGFR TKI-treated patients that are expressing this genetic polymorphism [141,142,143], and BH3 mimetics may overcome BIM polymorphism-associated TKI resistance [141].

## 6. Mechanisms of Resistance to Anti-EGFR Antibodies

mAbs (cetuximab and panitumumab) against EGFR have been clinically used as therapeutic drugs for CRC and head and SCCHN, and various resistant mechanisms also have been reported. Mutations in *KRAS* and *NRAS* in exon 2 are known to induce resistance to anti-EGFR antibodies in CRC [144,145,146]. For this reason, the expression of EGFR and wild-type KRAS is required for the use of these drugs, according to the FDA. In contrast, RAS mutations do not exclude these drugs from being used in patients with SCCHN because the relationship between the efficacy of these antibodies and RAS mutations has not been clarified [147]. To elucidate the mechanisms of resistance to anti-EGFR antibodies, whole-exome analysis of 129 tumors in patient-derived xenografts was performed for patients with CRC harboring wild-type KRAS [148]. Bypass signaling activation caused by gene amplification or mutations in RTKs (*HER2*, *FGFR1*, *MET*, and *PDGFR*), and the activation of downstream signaling pathways caused by mutations in *NRAS*, *BRAF*, or *PIK3CA*, or the deletion in *PTEN* were shown to contribute to resistance to anti-EGFR antibodies as alternative pathways. Moreover, AXL [149], VEGFR [150], MEK [151], and Src family kinase [152] have also been reported to mediate the activation of alternative pathways related to anti-EGFR antibody resistance. Notably, the variant form EGFRvIII, which lacks the extracellular domain, is found in less than 40% of SCCHN cases and induces resistance to cetuximab [153]. In patients with CRC, *EGFR* mutations (S492R [154], G465R, and G465E [148]) in exon 12, which encodes the extracellular domain of EGFR, are associated with resistance. Interestingly, among these mutations, the S429R mutation is known to obstruct binding of cetuximab to EGFR but does not inhibit the binding of panitumumab [154]. Alternatively, the overexpression of EGFR ligands (EGF, amphiregulin, and TGFα) [155], induction of the EMT [156], and enhancement of the nuclear translocation of EGFR [40] also contribute to anti-EGFR antibody resistance. Combination chemotherapies of anti-EGFR antibodies with EGFR TKIs [157,158,159], anti-HGF antibodies [160], and MEK inhibitors [151] are thought to overcome the resistance.

## 7. Conclusions

The use of EGFR TKIs or anti-EGFR mAbs for patients with NSCLC, CRC, pancreatic cancer, breast cancer, and SCCHN has improved patient prognosis in recent decades. However, these therapies are not able to completely cure the patients. Understanding these resistance mechanisms, which develop in cancer cells through inhibitor selection or changes to environmental conditions, will enable the establishment of therapies to overcome resistance. Decades of research has revealed multiple mechanisms modulating EGFR signals, and has resulted in the discovery of novel inhibitors that can suppress the development of resistant tumors. Analysis of the mechanisms modulating EGFR signaling and resistance to EGFR-targeted inhibitors has provided insights into the specific inhibition of EGFR signaling activity, e.g., by TKIs and mAbs, and has permitted further exploration of the development of drug-resistant cancers.

## Figures and Tables

**Table 1:** Epidermal growth factor receptor (EGFR) tyrosine kinase inhibitors approved for cancer treatment.

EGFR TKIs	Approved Indication	Target
Gefitinib (*Iressa*)	Metastatic non-small cell lung cancer (NSCLC) with *EGFR* exon 19 deletions or exon 21 mutation (L858R)	EGFR
Erlotinib (*Tarceva*)	Metastatic or locally advanced NSCNC, with EGFR exon 19 deletions or exon 21 mutation (L858R) Metastatic or advanced pancreatic cancer in combination with gemcitabine	EGFR, PDGFR, c-Kit
Afatinib (*Gilotrif*)	Metastatic NSCLC with *EGFR* exon 19 deletions or exon 21 mutation (L858R)	EGFR, HER2, HER4
Osimertinib (*Tagrisso*)	Metastatic *EGFR* T790M mutation-positive NSCLC, with progressive disease following first-line EGFR TKI therapy	EGFR T790M
Olmutinib (*Olita*)	Second-line treatment of NSCLC with the T790M mutation in *EGFR* (in Korea)	EGFR T790M
Lapatinib (*Tykerb*)	HER2-overexpressing breast cancer	EGFR, HER1, HER2
Vandetanib (*Caprelsa*)	Medullary thyroid carcinoma	EGFR, VEGFR, Ret

**Table 2:** Anti-EGFR monoclonal antibodies approved for cancer treatment.

EGFR-Targeted mAbs	Approved Indication	Target
Cetuximab (*Erbitux*)	Metastatic KRAS-negative CRC/SCCHN	EGFR
Panitumumab (*Vectibix*)	Metastatic KRAS-negative CRC	EGFR

## Abbreviations

EGFR	Epidermal growth factor receptor
ERBB	Avian erythroblastosis oncogene B
HER2	Human epidermal growth factor receptor 2
NSCLC	Non-small cell lung cancer
CRC	Colorectal cancer
SCCHN	Squamous cell carcinoma of the head and neck
PFS	Progress-free survival
TKI	Tyrosine kinase inhibitor
mAb	Monoclonal antibody
PDGFR	Platelet-derived growth factor receptor
NRG	Neuregulin
